# Utilizing the Metaverse for Learner-Centered Constructivist Education in the Post-Pandemic Era: An Analysis of Elementary School Students

**DOI:** 10.3390/jintelligence10010017

**Published:** 2022-03-07

**Authors:** Woong Suh, Seongjin Ahn

**Affiliations:** Department of Computer Education, Sungkyunkwan University, 25-2, Sungkyunkwan-Ro, Jongno-Gu, Seoul 03063, Korea; sjahn84@gmail.com

**Keywords:** metaverse, educational experience, attitude, constructivism, educational technology

## Abstract

Due to COVID-19, numerous new technologies are being implemented in education, with a growing interest in the metaverse. The term “metaverse” refers to an immersive digital environment where one can interact with virtual avatars. This study aims to analyze the experiences and attitudes of the metaverse for learner-centered education from a constructivist perspective to determine how closely related this virtual environment is to the lives of elementary school students. This study also examined how students are becoming the focal point of new educational technologies. After reviewing the literature on this topic, a survey of 336 elementary school students in Korea was conducted using 18 items for measuring each factor in the metaverse, followed by statistical analyses that included a difference of means and an independent sample *t*-test. The results revealed that, on average, 97.9% of elementary school students had experiences with the metaverse, with 95.5% of them considering it closely related to their everyday life. In addition, various conclusions according to each metaverse factor and each participant’s gender are provided.

## 1. Introduction

The virtual world (VW) is truly a Pandora’s box for educators (Kluge and Riley 2008). The metaverse—a compound term from the combination of transcendent (meta) and universe—is a science fiction world first mentioned in the novel *Snow Crash* (Stephenson 1993). It is a space that encompasses both reality and unreality across the various aspects of politics, economy, society, and culture. This study focuses on the educational aspects of the metaverse. Discussions regarding the metaverse, including virtual reality (VR), are becoming increasingly prevalent. For example, Roblox, an online game platform, has 199 million monthly users, with 54.86% of them being under the age of 13 years (Dean 2021). Consequently, because it has long since been established that educators must thoroughly understand their students to provide quality education, teachers need to understand that the metaverse is deeply embedded in their students’ lives to enhance their learning experience.

Educators often use technological resources to enhance the classroom learning experience (Gu et al. 2013). Below are several examples of the metaverse being used in education. First, it has been found that meaningful learning through the VW has the potential to provide numerous educational opportunities. One study in particular explored the various advantages and disadvantages of the VW in an academic environment with the aim of strengthening the curriculum using this technology (Kluge and Riley 2008; Manzoor 2017). Second, as a result of researching whether augmented reality (AR) technology can contribute to informing and educating elementary school students regarding the COVID-19 outbreak, it was found that it improves students’ cognitive abilities around knowledge retention, as well as their creativity. It has also been confirmed that autonomous learning can be developed using these technologies (Lopes and Gonçalves 2021). Moreover, in the field of history education, various cases of the term metaverse being used accurately have been studied, with the results then confirming that the metaverse can provide additional meaning to both teachers and educational policymakers (Choi and Kim 2017). Third, the application of an AR math game for elementary school students using MathBuilder (van der Stappen et al. 2019) and the use of an AR-supported storybook to reduce mathematical anxiety (Wangid et al. 2020) have also been studied.

However, rather than focusing on the education sector, these studies focus primarily on technologies such as VW and AR and the broader metaverse that includes them.

They primarily provide insights into the application of these technologies rather than those around the students themselves or the actual subject of education. However, from a constructivist perspective, the subject of learning should be the students themselves rather than the skills being taught (Polka 2001). Therefore, if educators are wanting to develop a better understanding of the relationship between students and the metaverse, implementing this technology in the educational process may pave the way for learner-centered constructivist education in the post-pandemic era. This is because, as per this theory, students process or construct new information by relating it to their experiences, attitudes, and beliefs (Clark 2018).

Considering these points, the following research questions were presented in terms of the students’ experiences and attitudes (including their beliefs):

Have students actually experienced the metaverse?

How positive are students’ attitudes toward the metaverse?

**Figure 1:**
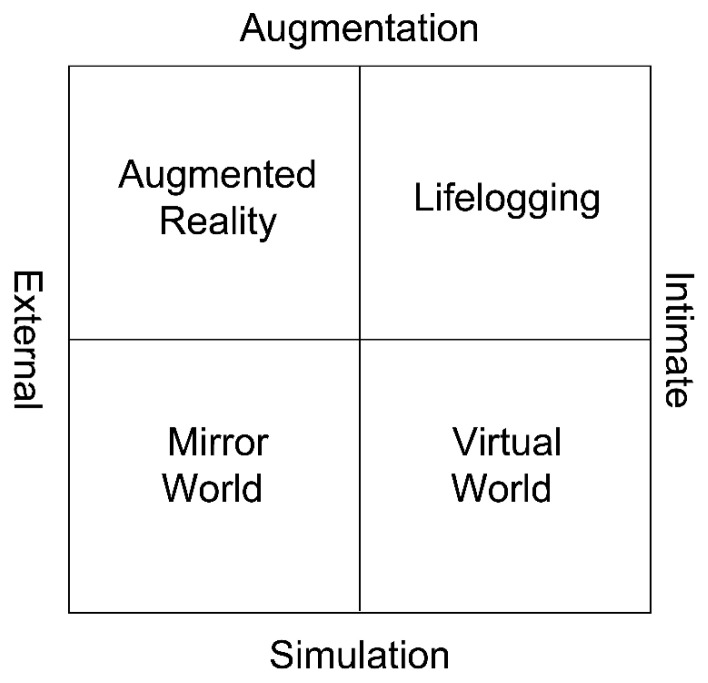
Metaverse classification (Smart et al. 2007).

Additionally, according to the four factors of the metaverse (see below in Figure 1), and considering the individual characteristics according to groups such as grade and gender, the following research question was also presented:

How do students’ experiences and attitudes manifest according to their individual grade and gender, as well as each factor of the metaverse?

Therefore, we aimed to discern whether utilizing the metaverse is appropriate for student-centered constructivist learning in the post-pandemic era, in addition to providing greater insights into this technology’s future educational uses by identifying each factor of the metaverse according to the participating student’s characteristics.

The metaverse, first mentioned in *Snow Crash* by Neal Stephenson, was later introduced to the public through a game called *Second Life*, created by Linden Labs in 2003. Since then, the metaverse has existed in various forms around us; however, interest in the metaverse has been increasing worldwide in recent years (Narin 2021). There are four reasons for this phenomenon. First, technological advancements, such as 5G and 3D rendering, have improved graphics, making the metaverse feel more real. Additionally, advanced Internet speeds have allowed people to enjoy the metaverse with no delays. Second, due to the ongoing COVID-19 pandemic, demand for non-face-to-face services has increased. Third, Generation Z—who are digital natives—have become more influential, resulting in changes in cultural consumption patterns. Fourth, the ubiquity of mobile devices and changes in content types have enabled people to access the metaverse anytime and anywhere (Ko et al. 2021). As communication technology, graphics, cloud computing, virtual reality, and artificial intelligence technologies have developed in an innovative fashion, it has become possible to configure a virtual space that is similar to reality and to provide it at a lower cost. If this was originally a “second” space concept, the recently developed metaverse could change it into a “first” space one that could even replace reality (Jeon 2021).

A more detailed example is the virtual concert held by musician Lil Nas X in November 2020 on Roblox, where more than 30 million viewers were recorded over a total of 4 performances (Millman 2020). In April 2021, Travis Scott held a virtual live concert in the game Fortnite with 27.7 million participants, including 5 performances of 10 min each (Goslin 2020). In addition, Naver’s avatar platform, ZEPETO, was launched in 165 countries in August 2018 and currently has 200 million subscribers worldwide, including in China, the United States, Japan, and Korea (Song 2021).

Specifically, the metaverse consists of a VW, a mirror world (MW), life logging (LL), and AR. A VW is a computer-simulated environment (Bartle 2004). A key component of the VW is one’s avatar (or in multiplayer games, character), the user’s personification in the VW. Examples of a VW include Roblox and Zepeto. A MW is an informationally enhanced virtual model or “reflection” of the physical world. Its construction involves sophisticated virtual mapping, modeling, and annotation tools, geospatial and other sensors, and location-aware and other LL (history-recording) technologies. Unlike the VW, which involves alternate realities that may be similar to Earth’s or wildly different, the MW models the world around us. The best-known example of a MW is presently Google Earth, a free, web-based, open-standards digital map of Earth. In AR, the metaverse enhances the external physical world for the individual using location-aware systems and interfaces that process and layer networked information on top of our everyday perception of the world. These include Pokemon Go or Snow (application), a type of selfie app. LL is the capture, storage, and distribution of everyday experiences and information for objects and people. It is represented by Instagram or Facebook, a social network service that many people enjoy joining (Smart et al. 2007; Figure 1).

These metaverses are expected to undermine the notions of race, gender, and even physical disability (Duan et al. 2021), which have provoked scholars’ and educators’ interest in them in terms of their potential use as a learning environment for various fields of education (Parmaxi 2020).

Moreover, studies on language learning, nursing education, and immersive VR apps, in addition to systematic reviews around the educational purposes of technologies like AR, have been conducted for examining their educational use (Akçayır and Akçayır 2017; Parmaxi 2020; Radianti et al. 2020). In relation to the subject of this study, research on elementary school students has shown that VR not only successfully augments science education but also engages students at all levels to make the learning environment equitable (Johnson et al. 2002). Another study showed that learning using AR has a positive effect on elementary school students (Lopes and Gonçalves 2021).

However, the aforementioned studies have one thing in common: the study situation was controlled for the purposes of the research. There has been no empirical study on whether students actually use the metaverse in a more natural environment. Therefore, we investigated a situation wherein students use the metaverse in a natural environment rather than in an experimental context for examining the general and universal educational use of the metaverse.

### Extended Technology Acceptance Model

The technology acceptance model (TAM) was developed as a theoretical framework to determine the factors influencing organizational members’ acceptance of information technology introduced to improve organizational performance (Davis 1989). The TAM investigates the issue of how users accept and use a specific technology as a function of the causal relationships among the system’s design features, its perceived usefulness (PUSF), its perceived ease of use (PEOU), the attitude towards its use, and its actual usage. The TAM assumes that user adoption and effective use are determined by one’s intention around using a given system, which in turn is affected by its perceived usefulness, ease of use, and the attitudes toward using it. Consequently, perceived usefulness and perceived ease of use are the two primary predictors of effective acceptance and use (Padilla-Meléndez et al. 2013).

According to Landry et al. (2006), the TAM is suitable for use in academic settings. Additionally, several factors, including the variables suggested by the TAM, should be investigated when examining whether users are satisfied with computer applications (Adams et al. 1992; Chau 1996, 2001). Therefore, to investigate the current research problem in relation to students’ attitudes, the extended TAM model was used. This model has an additional variable—perceived playfulness (PPLF)—that assists in explaining one’s interest and immersion in education (Moon and Kim 2001). Playfulness is an individual difference variable that allows people to frame or reframe everyday situations in such a way to experience them as entertaining, intellectually stimulating, or personally interesting (Proyer 2017). When learning activities become interesting, students will continue to improve and excel in their education. This is because past evidence has suggested that one’s degree of interest is related to their performance in the academic context (Nye et al. 2012). Examples of this model being used for educational research include cases in which the difference in PPLF according to gender in a blended learning scenario was determined, and studies that investigated the role of PPLF in learning management systems were conducted (Balkaya and Akkucuk 2021; Padilla-Meléndez et al. 2013).

**Figure 2:**
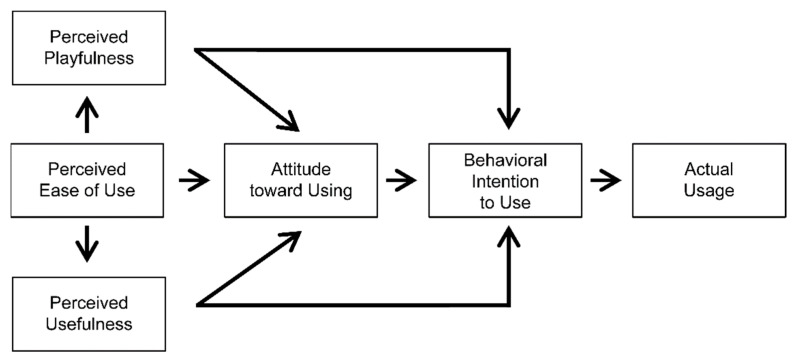
Extended TAM model (Moon and Kim 2001).

The extended TAM model consists of PPLF, PEOU, PUSF, the attitude toward use (ATT), behavioral intention (BI) to use, and actual usage. Strictly speaking, the attitude mentioned in the above models only refers to ATT, but in this study, when “attitude” is mentioned later, all five variables related to PPLF, PEOU, PUSF, ATT, and BI were placed into the ATT category. In this study, actual usage was divided into another questionnaire: experience, frequency of use (FR), and use time (UT; Figure 2).

## 2. Materials and Methods

### 2.1. Respondents

For this study, a survey was conducted among 336 elementary school students in Korea from June to July 2021. This study was approved by the Institutional Review Board (IRB) of Sungkyunkwan University (approval number SKKU2021-06-021). Written informed consent was obtained from all the students’ parents. As informatics is an assigned course in the fifth and sixth grades in the Korean public curriculum, 217 fifth graders and 119 sixth graders (165 males and 171 females) were sampled in this study, as depicted in Table 1 below.

### 2.2. Measurement Development

Students process or construct new information by relating it to their experiences, attitudes, and beliefs (Clark 2018). Therefore, items for examining the research questions consisted of those that measured respondents’ experiences and attitudes.

Because the respondents were elementary school students, the questions about their experiences included “The mirror world means a metaverse created by taking and copying the shape, information, and structure of the real world. Examples: Zoom, Google Earth, etc.” Regarding their experience, the presence or absence of any experience for each factor was collected as either “yes” or “no”. The FR question was “How often do you use the virtual world during a week?” The possible answers were as follows: “not at all”, “less than once a week”, “about once a week”, “two to three times a week”, “several times a week”, “about once a day”, and “several times a day”. Information about UT was collected using the question “How long do you use the virtual world in a week?” Respondents chose from several options, including “not at all”, “under 1 h”, “between 1 and 5 h”, “between 5 and 10 h”, “between 10 and 15 h”, “between 15 and 20 h”, and “over 20 h”. The questions on respondents’ attitudes were adapted and modified by referring to the questions presented in the extended TAM model of Moon and Kim (2001) and were composed of PPLF, PEOU, PUSF, ATT, and BI. Three questions were investigated for each construct, with the respondents’ degree of agreement with each question being investigated using a 5-point Likert scale (Table 2).

## 3. Results

### 3.1. Statistical Results

#### 3.1.1. Experience

Our statistical and analytical results for the following items were calculated from the results of the experience, FR, and UT measures for each factor of the metaverse as follows:

- Whether the respondent had experienced each simple factor;
- The number of respondents with overlapping experiences among the metaverse factors;
- The number of experienced persons per each metaverse factor for the respondents as divided by grade and gender (the ratio of experienced persons to the number of respondents by item was calculated using a chi-square test);
- The ratio of FR to experienced users by factor;
- The ratio of UT to experienced users by factor.

The significance test and its findings for FR and UT by gender were found using an independent sample *t*-test.

#### 3.1.2. Attitude

By collecting the responses to PPLF, PEOU, PUSF, ATT, and BI for each factor in the metaverse, statistics and analysis results of the following items were calculated:

- The mean and standard deviation of the respondents’ attitude components for each construct;
- The significance of the attitude variables by grade and gender (using an independent sample *t*-test).

### 3.2. Confirmation of Reliability and Validity of the Model Used in the Study

To confirm the reliability of the items used, reliability analysis was performed on the constructs constituting the attitude of each factor in the metaverse. Validity was verified through confirmatory factor analysis (CFA). The results of the analysis on whether the items and models were appropriate can be found in Table 3 and Table 4.

#### 3.2.1. Reliability Analysis

Generally, the reliability is considered good if it is 0.7 or higher. The Cronbach’s alpha coefficient was calculated by grouping three items constituting each component, all of which were higher than 0.7. This indicated that the reliability of the main items of this study was good. Therefore, no items impaired the model’s reliability, with the analysis then being conducted without removing any of the items (Table 3).

#### 3.2.2. Validity Analysis

A CFA was performed to confirm the validity of the items. Validity is secured when the standardized λ value is 0.5 or higher, the average variance extracted (AVE) is 0.5 or higher, and the construct reliability (CR) is 0.7 or higher. After performing a CFA on the sub-factors for each component, standardized and appropriate λ, AVE, and CR values were all secured, as illustrated in Table 4. Therefore, the model used in the study was valid.

#### 3.2.3. Experience Analysis

The respondents were asked whether they had any experience with each factor of the metaverse through questions such as “Have you ever used a metaverse related to the virtual world?” Of the respondents, 306 (91.1%) answered that they had experiences with the VW, 277 (82.4%) with the MW, 185 (55.1%) with LL, and 252 (75%) with AR (Table 5).

Based on the above responses, the analytical results of the overlapping experiences between the elements showed that 136 students (40.5%) had experienced all four metaverses, 76 students (22.6%) had experienced the VW, the MW, and AR but had not experienced LL, 33 students (9.8%) had experienced the VW and the MW but had not experienced LL or AR, and 7 students (2.1%) had not experienced any of them. The number and rate of all other responses are collated in Table 6.

#### 3.2.4. FR and UT Analysis

The results of asking the students who had experienced each factor of the metaverse about their FR and UT can be found in Table 7 and Table 8.

In the case of the VW, the response of “several times each day” was the highest among all factors (22.5%), which was followed by LL (21.6%). Conversely, 43.7% of the students who had used AR answered that they had used it very rarely. In terms of UT, in the case of the VW and the MW, the percentage of students who responded with more than 1 h to less than 5 h for each item was the highest (VW: 37.6%, MW: 38.6%), and in the case of LL and AR, the percentage of students who answered yes (LL: 54.6%, AR: 71.0%) was the highest for the less than 1 h option.

An independent sample *t*-test was performed to verify whether there was a significant difference in FR and UT based on gender. LL FR showed a significant difference according to gender (t = −5.093, *p* < 0.001), with the values for females (M = 4.71) being higher than that for males (M = 3.33). Furthermore, LL UT also showed a significant difference according to gender (t = −2.092, *p* < 0.05), with it being found that females (M = 2.87) had higher scores here than males (M = 2.44).

#### 3.2.5. Attitude Analysis

The average of the attitude components for each factor is the average of the results for the students who experienced each factor of the metaverse for PPLF, PEOU, PUSF, ATT, and BI, and the results for this are exhibited in Table 9.

All the mean values were between three and four points. On average, the item with the highest score for all factors was PEOU (M = 3.99), with the lowest score being that for PUSF (M = 3.21).

For analysis of the significance of the constructs of the attitude toward each factor of the metaverse according to grade and gender, an independent sample *t*-test was conducted to verify whether the constructs of the attitude toward each metaverse factor showed a significant difference according to the respondents’ grade and gender. The results, verified for significance, are presented in Table 10 below.

First, in the case of the VW, depending on gender, VW_PPLF (t = 4.539, *p* < 0.001, male (M) = 4.20, female (M) = 3.78), VW_ATT (t = 2.569, *p* < 0.05, male (M) = 3.61, female [M] = 3.34) and VW_BI (t = 2.310, *p* < 0.05, male (M) = 3.59, female (M) = 3.32) had a significant difference found between male and female students for all three variables. In the case of AR, AR_PPLF (t = 2.867, *p* < 0.01) for male students (M = 3.88) showed significantly higher results than that for female students (M = 3.51) for one construct. As a result of verifying whether the differences in the constructs of attitudes toward LL were significant, we found LL_PPLF to differ significantly according to gender (t = −2.910, *p* < 0.01), with females (M = 3.95) having higher results than their male counterparts (M = 3.50). This was also the case for LL_BI (t = −1.657, *p* < 0.05), with females scoring higher (M = 3.79) than males (M = 3.37).

Some variables also showed significant differences according to grade. For example, LL_PEOU was higher among the sixth grade respondents (M = 4.18) than among the fifth grade ones (M = 3.87; t = −2.174, *p* < 0.05). LL_BI also showed a significant difference according to grade (t = −2.293, *p* < 0.05), with us finding that the sixth grade respondents (M = 3.89) scored higher here than the fifth grade ones (M = 3.50). No significant difference was found for all constructs that were not presented herein.

## 4. Discussion

### 4.1. Experience

When responding to our first research question, (“Have students actually experienced the metaverse?”) 327 out of 336 respondents (97.9%) answered that they had experienced at least one metaverse. In the case of the VW, 91.1% of the students answered that they had experienced it. In the case of LL, the lowest factor, more than half of the students (55.1%) answered that they had experienced it. These findings are significant, because this is the first empirical study to confirm that surveyed elementary school students actually used the metaverse in natural situations (i.e., in their everyday lives) and not only within experimental conditions.

### 4.2. Frequency of Use and Use Time

Pursuant to surveying students who had experienced each factor of the metaverse regarding their FR and UT, on average, only 4.5% of the students answered “Not at all”. In the case of FR, the cumulative response for use more than once a week reached 81.0%, 83.3%, and 74.0% for the VW, the MW, and LL, respectively. The cumulative response that stated that UT was used more than once a week was 63.0% for the VW and 69.2% for the MW. These results reveal that the metaverse occupied a significant proportion of the lives of the fifth and sixth graders at the elementary school being investigated.

Specifically, in the case of the MW, “two or three times a week” (27.8%) and “1–5 h” (38.5%) accounted for the largest number of students. This was likely affected by the FR and UT of online classes currently held in public schools in Korea, which could be a sign of changing times wherein metaverses are becoming required experiences (even considering the special circumstances caused by COVID-19). In addition, in the case of LL, the FR of “several times each day” was the highest (21.6%), with the UT of “under 1 h” (52.4%) being the highest. It is likely that the respondents’ behaviors in regard to social network services (SNSs), which are easily accessed when time permits in a way that most often involves checking whether there are new messages or posts on one’s feed and which then ends immediately thereafter, influenced the statistical results around the LL. In addition, LL showed a significant difference according to gender in FR and UT. In the case of FR, the female students showed significantly higher FR values (M = 4.71) than the male students. Similarly, in terms of UT, the female students (M = 2.87) scored significantly higher than their male counterparts (M = 2.44). If we consider this analysis together with the significance test results of the differences in the LL variables according to grade and gender, it is revealed that the female students thought that LL was interesting and had a greater intention to use it than the male students did. Moreover, the female students experienced more LL than the male ones did (29.6%) and were active more often and for a longer duration therein. In addition, it was confirmed that the sixth graders thought it was easier to use LL than the fifth graders did, with them then having greater intentions to use them simultaneously.

### 4.3. Attitude

For the VW, the PPLF score was the highest among the four constructs (3.99), with PUSF being the lowest (3.03). Conversely, in the case of the MW, we found that PPLF was the lowest at 3.40, with PUSF being the highest at 3.61. In the case of the MW, the primary reason for its use seemed to be for online classes represented by Zoom. These results revealed that the participating students agreed to some extent on the usefulness of online classes but did not think of them as fun. The mean of these PPLF scores for the MW, which was significantly lower than that of the other three factors, revealed that our focus needs to go beyond simply applying new technologies such as the metaverse to education; rather, both teachers and researchers need to begin undertaking constructivist education that allows students to become both immersed and interested. However, the students considered the VW to be fun because they were exposed to it as a form of entertainment with games like Roblox and Minecraft, but they seemed to have doubts about its usefulness. Therefore, if teachers know that a VW can be useful to students and use it educationally, positive effects can be expected due to the favorable attitude that students have toward it. Additionally, in the case of the VW, it was confirmed that the male students had significantly higher scores than the female students in terms of PPLF, ATT, and BI.

Furthermore, considering that LL and AR also had relatively high PPLF but low PUSF averages, it is necessary to have students recognize that metaverses such as the VW, LL, and AR are useful in their lives before applying them to education.

Conversely, the highest average item among the four factors of the metaverse was PEOU (M = 3.99), which implied that students do not experience technical difficulties in using any of the studied metaverses, a result that concurs with Prensky’s (2001) definition of digital natives.

## 5. Conclusions

This study primarily analyzed the experiences of elementary school students regarding their use of the metaverse to determine whether it is suitable for learner-centered constructivist education in the post-pandemic era.

The results confirm that almost all of the respondents (97.9%) had prior experiences with using the metaverse. In addition, the survey on the FR and UT of the students who had metaverse experiences found that, although each metaverse factor was slightly different, on average, 95.5% of the students continued to use the metaverse after their initial experiences. After combining these results, although there are slight differences by factor, grade, and gender, the metaverse appears to be closely related to the lives of elementary school students. It has been stated that education should be learner-centered, according to the constructivist perspective (Fosnot 2013), with the results derived from this study statistically demonstrating that the educational use of the metaverse can benefit most students.

The second research question of this study was to determine how elementary school students’ experiences and attitudes were demonstrated according to each metaverse factor, grade, and gender. In the case of the VW, it was found to be relatively fun but not useful, and in the case of the MW, it was not found to be fun but was useful. Therefore, in the case of the VW, it is necessary to have content that gives the perception that it is useful for students’ lives, and in the case of the MW, a teaching method that can provide them with more fun and immersion is needed.

In the case of LL, it was confirmed that the female students had a more positive attitude. More specifically, in the case of FR, it was confirmed that using LL “several times each day” was common, being at a rate of 21.6%. However, in UH, as displayed in Table 8, “under 1 h” was found to be less common (54.6%). Moreover, LL_PEOU was higher in the sixth grade students (M = 4.18) than in the fifth graders (M = 3.87; t = −2.174, *p* < 0.05). LL_BI also showed a significant difference according to grade (t = −2.293, *p* < 0.05), with us finding that the sixth grade students (M = 3.89) scored higher than those in the fifth grade (M = 3.50). These data provide several implications. First, the elementary-school-aged female students had a positive attitude toward LL, as represented by Facebook or Instagram. Second, the data revealed that LL was frequently used but not for long periods of time. Third, the higher the respondent’s grade, the higher their interest in and their intention of using LL was.

Until now, through various studies, the low participation of women in science, technology, engineering, and mathematics (STEM) studies has been identified as one of the main problems that must be resolved to reduce the gender gap that exists in the technology sector (García-Holgado et al. 2018; Virtanen et al. 2015). To solve this problem, there have been several studies on the educational use of SNS, which is a representative of LL. However, the samples of these studies included students receiving higher education (Akçayir 2017; Torun 2019, 2020). As such, our finding that elementary school female students had a positive attitude toward LL is noteworthy in that it suggests a way to solve the gender gap in the technology sector. Thus, policymakers, school administrators, and teachers should consider using LL, especially among the available metaverse factors, to narrow the gender gap in the technology sector. In addition, because PEOU had the highest score among the four metaverse factors, this indicates that the students who had not yet experienced each factor of the metaverse could use it without difficulty if there was an appropriate opportunity. This also shows the possibility and necessity of research on whether the metaverse can be an appropriate educational environment for children with special needs, such as those with autism or physical disabilities. This is because SNSs and other online communities are an appropriate educational environment for children who are physically or emotionally unable to adapt to lessons conducted in classrooms (Kurniawati et al. 2019; Ringland et al. 2016; Stewart Rosenfield et al. 2019). However, for the effective educational use of the metaverse, more diverse studies should be conducted in the future. Several other factors should be considered when using the metaverse in education in addition to students’ experience and attitude. For example, in gamified education, posting current scores is suitable for introverts, with the posting of rewards being more appropriate for extroverts (Codish and Ravid 2014). Further research can be conducted on various other aspects that should be considered while implementing the use of the metaverse in education.

On the other hand, it should be noted that this study has several limitations. First, it only included the fifth and sixth graders of an elementary school in Korea. From a regional standpoint, it is necessary to consider that the respondents to this study are more likely to have access to the metaverse than other regions of the world. In other words, a student in a location without a stable Internet connection will not have the same level of experience or knowledge as the participants of this study. Second, regarding the educational level of the respondents, there is a need to study perceptions in a wider range of grades and populations. If this is performed, researchers will be able to obtain more meaningful data and draw conclusions about a wider variety of students (i.e., not only elementary school students but also middle and high school students, college students, and the general public). Finally, the responses used in this study were self-reported data of students’ opinions and may not have been representative of the actual behavior of the students. The respondents may not have fully understood the concepts or may have miscategorized their experiences. According to Polka (1999), educational technologies were expected to profoundly impact curricula and teaching and learning methodologies in the new millennium. Since that study, more than 20 years on, the technologies surrounding our students have been evolving on a daily basis, and the process of including technology within the classroom context is inexhaustible and goes hand in hand with the evolution of technology (Toto and Limone 2021a). In this environment, educators have a role of renewed authority because, now aware of their own abilities and limitations, they exploit the digital resources available to build a strong educational process (Toto and Limone 2021b). However, efforts to understand these technologies from a student’s point of view rather than a technical one have been neglected. To understand the perspective of a student, educators must also understand the world in which they live as well as their interests. When working with students, it is more important to understand what technology they are using and how it affects their growth and relationships rather than only focusing on how much time is spent on it (Turner 2015). Thus, the results of this study will be helpful in understanding students and will provide a theoretical and academic foundation for using the metaverse for educational purposes.

## Figures and Tables

**Table 1:** Respondents’ profile.

	Fifth Grade	Sixth Grade	Sum
Male	110	55	165
Female	107	64	171
Sum	217	119	336

**Table 2:** Examples of survey questions and research tools.

Classification	Factor	Examples	Number
Experience	Exp.	Have you ever used a virtual world?	1
	FR	How often do you use the virtual world in a week?	1
	UT	How long do you use the virtual world in a week?	1
Attitude	PPLF	It is fun to use the mirror world.	3
	PEOU	Learning to use the mirror world is easy.	3
	PUSF	Learning using the mirror world is helpful to me.	3
	ATT	Using mirror world is a good idea.	3
	BI	I will continue to use the mirror world in the future.	3

FR: frequency of use; UT: use time; PPLF: perceived playfulness; PEOU: perceived ease of use; PUSF: perceived usefulness; ATT: attitude toward use; BI: behavioral intention.

**Table 3:** Reliability analysis for each question (Cronbach’s alpha).

	VW	MW	LL	AR	Number of Question
PPLF	0.780	0.796	0.870	0.852	3
PEOU	0.785	0.847	0.900	0.834	3
PUSF	0.857	0.808	0.848	0.892	3
ATT	0.816	0.833	0.883	0.844	3
BI	0.880	0.870	0.884	0.916	3

VW: virtual world; MW: mirror world; LL: life logging; AR: augmented reality; PPLF: perceived playfulness; PEOU: perceived ease of use; PUSF: perceived usefulness; ATT: attitude toward use; BI: behavioral intention.

**Table 4:** Results of validation of usage model.

Factor			S.E.	C.R.	Standardized Coefficient	AVE	Construct Reliability
VW_PPLF3	<-	VW_PPLF			0.666	0.567	0.796
VW_PPLF2	<-	VW_PPLF	0.084	11.990	0.824		
VW_PPLF1	<-	VW_PPLF	0.083	11.567	0.783		
VW_PEOU3	<-	VW_PEOU			0.836	0.588	0.809
VW_PEOU2	<-	VW_PEOU	0.074	12.339	0.751		
VW_PEOU1	<-	VW_PEOU	0.066	10.716	0.643		
VW_PUSF3	<-	VW_PUSF			0.793	0.609	0.824
VW_PUSF2	<-	VW_PUSF	0.066	15.749	0.846		
VW_PUSF1	<-	VW_PUSF	0.065	15.026	0.811		
VW_ATT1	<-	VW_ATT			0.728	0.562	0.794
VW_ATT2	<-	VW_ATT	0.081	13.622	0.779		
VW_ATT3	<-	VW_ATT	0.080	13.971	0.798		
VW_BI1	<-	VW_BI			0.867	0.666	0.856
VW_BI2	<-	VW_BI	0.048	22.155	0.917		
VW_BI3	<-	VW_BI	0.052	16.322	0.767		
MW_PPLF3	<-	MW_PPLF			0.635	0.542	0.778
MW_PPLF2	<-	MW_PPLF	0.119	11.085	0.871		
MW_PPLF1	<-	MW_PPLF	0.117	10.647	0.804		
MW_PEOU3	<-	MW_PEOU			0.789	0.656	0.851
MW_PEOU2	<-	MW_PEOU	0.072	14.090	0.861		
MW_PEOU1	<-	MW_PEOU	0.078	13.095	0.780		
MW_PUSF3	<-	MW_PUSF			0.738	0.540	0.779
MW_PUSF2	<-	MW_PUSF	0.075	13.000	0.787		
MW_PUSF1	<-	MW_PUSF	0.073	12.880	0.780		
MW_ATT1	<-	MW_ATT			0.815	0.613	0.826
MW_ATT2	<-	MW_ATT	0.060	16.527	0.829		
MW_ATT3	<-	MW_ATT	0.070	14.560	0.759		
MW_BI1	<-	MW_BI			0.816	0.657	0.851
MW_BI2	<-	MW_BI	0.058	18.087	0.901		
MW_BI3	<-	MW_BI	0.064	15.137	0.795		
LL_PPLF3	<-	LL_PPLF			0.780	0.645	0.844
LL_PPLF2	<-	LL_PPLF	0.073	13.916	0.912		
LL_PPLF1	<-	LL_PPLF	0.072	12.399	0.831		
LL_PEOU3	<-	LL_PEOU			0.872	0.744	0.897
LL_PEOU2	<-	LL_PEOU	0.062	16.583	0.901		
LL_PEOU1	<-	LL_PEOU	0.065	14.579	0.834		
LL_PUSF3	<-	LL_PUSF			0.738	0.537	0.776
LL_PUSF2	<-	LL_PUSF	0.094	11.690	0.886		
LL_PUSF1	<-	LL_PUSF	0.098	10.340	0.778		
LL_ATT1	<-	LL_ATT			0.958	0.734	0.891
LL_ATT2	<-	LL_ATT	0.057	16.791	0.879		
LL_ATT3	<-	LL_ATT	0.061	12.289	0.727		
LL_BI1	<-	LL_BI			0.905	0.641	0.842
LL_BI2	<-	LL_BI	0.054	18.078	0.879		
LL_BI3	<-	LL_BI	0.068	13.976	0.777		
AR_PPLF3	<-	AR_PPLF			0.738	0.598	0.816
AR_PPLF2	<-	AR_PPLF	0.075	13.972	0.887		
AR_PPLF1	<-	AR_PPLF	0.073	13.402	0.849		
AR_PEOU3	<-	AR_PEOU			0.819	0.647	0.845
AR_PEOU2	<-	AR_PEOU	0.075	14.023	0.879		
AR_PEOU1	<-	AR_PEOU	0.067	11.317	0.691		
AR_PUSF3	<-	AR_PUSF			0.855	0.647	0.846
AR_PUSF2	<-	AR_PUSF	0.054	16.675	0.852		
AR_PUSF1	<-	AR_PUSF	0.053	17.067	0.866		
AR_ATT1	<-	AR_ATT			0.729	0.606	0.820
AR_ATT2	<-	AR_ATT	0.109	12.279	0.919		
AR_ATT3	<-	AR_ATT	0.097	11.702	0.773		
AR_BI1	<-	AR_BI			0.940	0.695	0.872
AR_BI2	<-	AR_BI	0.040	24.640	0.907		
AR_BI3	<-	AR_BI	0.048	19.191	0.822		

**Table 5:** Experience by factor.

	Experienced	Inexperienced	Sum
VW	306 (91.1%)	30 (8.9%)	336 (100%)
MW	277 (82.4%)	59 (17.6%)	
LL	185 (55.1%)	151 (44.9%)	
AR	252 (75%)	84 (25%)	

VW: virtual world; MW: mirror world; LL: life logging; AR: augmented reality.

**Table 6:** Number of respondents with overlapping experiences between metaverse factors.

		VW (E)		VW (I)	
		LL (E)	LL (I)	LL (E)	LL (I)
MW (E)	AR (E)	136 (40.5%)	76 (22.6%)	6 (1.8%)	6 (1.8%)
	AR (I)	16 (4.8%)	33 (9.8%)	2 (0.6%)	2 (0.6%)
MW (I)	AR (E)	11 (3.3%)	13 (3.9%)	2 (0.6%)	2 (0.6%)
	AR (I)	9 (2.7%)	12 (3.6%)	3 (0.9%)	7 (2.1%)

VW: virtual world; MW: mirror world; LL: life logging; AR: augmented reality; E: experienced; I: inexperienced.

**Table 7:** Ratio of FR to experienced persons by factor.

FR	VW	MW	LL	AR
Not at all	2.6%	1.4%	5.4%	8.7%
Less than once a week	16.3%	15.2%	20.5%	43.7%
About once a week	11.4%	9.0%	15.1%	16.7%
2 or 3 times a week	20.3%	27.8%	17.3%	13.5%
Several times a week	16.7%	24.9%	10.3%	8.7%
About once a day	10.1%	12.6%	9.7%	5.2%
Several times each day	22.5%	9.0%	21.6%	3.6%
Sum	100%	100%	100%	100%

FR: frequency of use; VW: virtual world; MW: mirror world; LL: life logging; AR: augmented reality.

**Table 8:** Ratio of UH to experienced persons by factor.

UT	VW	MW	LL	AR
Not at all	2.6%	1.4%	5.4%	8.7%
Under 1 h	34.3%	29.2%	54.6%	71.0%
1–5 h	37.6%	38.6%	22.2%	13.5%
5–10 h	10.1%	14.1%	7.0%	3.6%
10–15 h	5.2%	9.7%	3.2%	1.2%
15–20 h	6.5%	3.6%	4.3%	1.2%
Over 20 h	3.6%	3.2%	3.2%	0.8%
Sum	100%	100%	100%	100%

UT: use time; VW: virtual world; MW: mirror world; LL: life logging; AR: augmented reality.

**Table 9:** Average of attitude components for each factor.

	PPLF		PEOU		PUSF		ATT		BI		Sum
	M	Std.	M	Std.	M	Std.	M	Std.	M	Std.	
VW	3.99	0.84	4.05	0.80	3.03	1.00	3.47	1.05	3.45	0.91	306
MW	3.40	0.95	3.93	0.87	3.61	0.94	3.66	0.99	3.69	0.92	277
LL	3.79	1.02	3.99	0.95	3.17	1.09	3.82	1.10	3.64	0.93	185
AR	3.68	1.04	4.02	0.87	3.05	1.12	3.62	1.19	3.37	0.98	285
Average	3.71		3.99		3.21		3.64		3.54		

VW: virtual world; MW: mirror world; LL: life logging; AR: augmented reality; PPLF: perceived playfulness; PEOU: perceived ease of use; PUSF: perceived usefulness; ATT: attitude toward use; BI: behavioral intention.

**Table 10:** Significance verification of attitude constructs by grade and gender.

		Group	Samples	Average	Std.	t	*p*
Gender	VW_PPLF	M	150	4.20	0.72	4.539 ***	0
		F	156	3.78	0.89		
	VW_ATT	M	150	3.61	0.86	2.569 *	0.011
		F	156	3.34	0.94		
	VW_BI	M	150	3.59	1.01	2.310 *	0.022
		F	156	3.32	1.07		
	AR_PPLF	M	116	3.88	1.05	2.867 **	0.004
		F	136	3.51	1.01		
	LL_PPLF	M	66	3.50	1.12	−2.910 **	0.004
		F	119	3.95	0.93		
	LL_BI	M	66	3.37	1.13	−2.540 *	0.012
		F	119	3.79	1.06		
Grade	LL_PEOU	5g	118	3.87	0.97	−2.174 *	0.031
		6g	67	4.18	0.88		
	LL_BI	5g	118	3.50	1.05	−2.293 *	0.023
		6g	67	3.89	1.16		

* *p* < 0.05. ** *p* < 0.01. *** *p* < 0.001.

## Data Availability

The data presented in this study are available on request from the corresponding author. The data are not publicly available due to privacy.
